# Functional Integration of a Portable Non-Enzymatic Electrochemical Glucose Sensor in Simulation-Based Medical Education Through a Teleconsultation Workflow

**DOI:** 10.3390/s26092787

**Published:** 2026-04-30

**Authors:** Leonel Vasquez-Cevallos, Darwin Castillo, Pedro A. Salazar-Carballo, Paul E. D. Soto-Rodriguez, Franklin Parrales-Bravo, Victor H. Guarochico-Moreira, Roberto Tolozano-Benites

**Affiliations:** 1 Facultad de Ingenierías, Arquitectura y Ciencias de la Naturaleza, Universidad Ecotec, Samborondón 092302, Ecuador; 2 Departamento de Química, Facultad de Ciencias Exactas y Naturales, Universidad Técnica Particular de Loja, Loja 110107, Ecuador; 3 Laboratory of Sensors, Biosensors and Advanced Materials, Institute of Materials and Nanotechnology, University of La Laguna (ULL), 38200 La Laguna, Spain; psalazar@ull.edu.es; 4 Instituto de Estudios Avanzados IUDEA, Departamento de Física, Universidad de La Laguna, 38203 Tenerife, Spain; psotorod@ull.edu.es; 5 Faculty of Computer Science, Complutense University of Madrid, 28040 Madrid, Spain; fparrale@ucm.es; 6 Centro de Estudios en Tecnologías Aplicadas, Universidad Bolivariana del Ecuador (UBE), Durán 092405, Ecuador; rtolozano@ube.edu.ec; 7 Facultad de Ciencias Naturales y Matemáticas, Centro de Investigación y Desarrollo en Nanotecnología, Escuela Superior Politécnica del Litoral (ESPOL), Guayaquil 090902, Ecuador; vhuguaro@espol.edu.ec

**Keywords:** non-enzymatic glucose sensor, portable sensor, copper-modified electrode, electrochemical sensing, telemedicine, teleconsultation, simulation-based medical education

## Abstract

Portable non-enzymatic electrochemical glucose sensors offer potential for decentralized healthcare and medical education; however, their integration into simulation-based teleconsultation training workflows remains limited. This study presents the functional integration of a portable copper-modified electrochemical glucose sensor into a web- and Android-based telemedicine platform within a simulation-based medical education framework. Screen-printed carbon electrodes were electrochemically activated and modified via copper electrodeposition. Surface and electrochemical characterization were performed using SEM-EDX and cyclic voltammetry, respectively, followed by chronoamperometry for quantitative detection. Glucose solutions in PBS (pH 10) were measured using 70 µL samples, and the resulting signals were converted into glucose values (mg/dL) through a calibration model and incorporated into simulated gynecological teleconsultation workflows. The sensor exhibited a stable amperometric response at +0.60 V, with a linear range of 3.125–50 mM (R^2^ = 0.9822), an area-normalized sensitivity of 0.061 µA·mM^−1^·cm^−2^, and a limit of detection of 1.39 mM. Implementation within the simulation scenario (*n* = 26) demonstrated 69% high/very high perceived usability and 88% high/very high educational value. These results support the feasibility of functionally integrating portable electrochemical sensing into simulation-based teleconsultation training and provide a proof-of-concept framework for future technical refinement and broader educational validation.

## 1. Introduction

Diabetes is a major contributor to global mortality, with approximately 3.4 million deaths reported in 2024 [1,2]. This burden is particularly significant in developing countries, such as Ecuador, where limited healthcare resources and systemic constraints hinder effective disease management [3,4]. Meanwhile, undergraduate students require safe and structured training environments to develop practical skills and clinical decision-making in diabetes-related care [5]. Consequently, there is a growing need for accessible and cost-effective glucose monitoring solutions that can support both decentralized healthcare delivery and health science education settings [6].

In this context, electrochemical sensing platforms enable portable glucose detection through the integration of miniaturized electrodes and portable potentiostats [7,8,9]. A key advancement is the transition from enzymatic to non-enzymatic sensors, as enzymatic approaches are constrained by the instability of glucose oxidase [10]. Non-enzymatic sensors rely on the direct oxidation of glucose at electrocatalytic surfaces, including nanostructured transition metal oxides, noble metals, and carbon-based materials [11]. Among these, carbon electrodes can be electrochemically activated via cyclic voltammetry and subsequently modified through copper electrodeposition, generating redox-active sites that enhance glucose oxidation [12,13]. Copper-based modification strategies are particularly attractive because of their electrocatalytic activity, relatively low material cost, and compatibility with screen-printed electrode formats suited to portable devices. In contrast to more complex nanostructured architectures, direct copper electrodeposition on commercially available screen-printed carbon electrodes offers a simpler route for proof-of-concept integration into educational prototypes and teleconsultation-oriented engineering workflows. The resulting electrochemical response is commonly characterized using cyclic voltammetry and chronoamperometry, with glucose concentration quantified through linear calibration models [13,14].

Despite these advancements, the practical adoption of portable electrochemical sensors within telemedicine platforms and simulation-based environments remains challenging because of the limited seamless integration of sensor outputs into clinical and educational workflows [15,16,17]. This challenge is compounded by the need for efficient conversion of raw electrochemical signals converted into glucose values interpretable within a simulated teleconsultation workflow [18], which often requires additional post-processing to obtain quantitative glucose values [8,19].

While our previous work validated the web- and Android-based telemedicine platform for educational and rural healthcare use, the present study focuses on the functional incorporation of a portable electrochemical glucose sensing module into that workflow under simulation-based conditions.

Building upon these identified gaps, this study aimed to evaluate the feasibility and practical integration of a portable copper-modified non-enzymatic electrochemical glucose sensor into a web- and Android-based telemedicine platform. Specifically, it investigated whether electrochemical signals could be reliably converted into clinically interpretable glucose values (mg/dL) and seamlessly incorporated into a teleconsultation workflow for use in simulated gynecological scenarios.

## 2. Materials and Methods

### 2.1. Portable Electrochemical Sensor Platform

**Figure 1:**
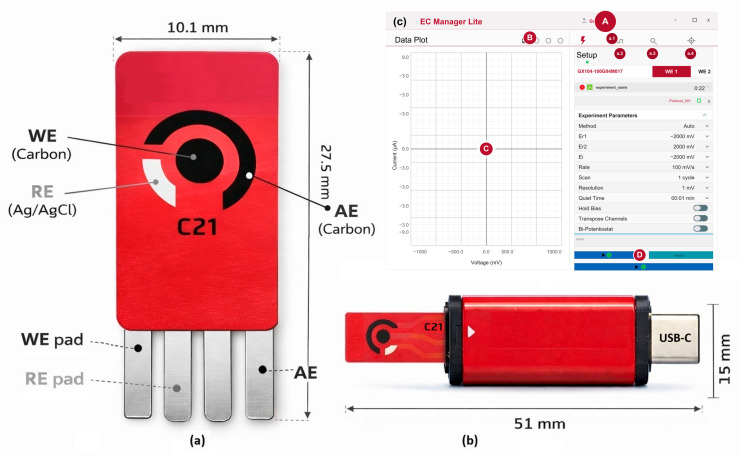
Electrochemical sensing platform: (**a**) Screen-printed carbon electrode (ED-S1PE-C21). (**b**) Portable ECSens bipotentiostat. (**c**) Graphical User Interface (GUI) of EC Manager Lite for Windows PC.

Screen-printed carbon electrodes (ED-S1PE-C21, MicruX Technologies, Gijón, Spain) were used for all electrochemical experiments. Each strip integrates a carbon working electrode (3 mm diameter; 7.1 mm^2^ geometric area), a carbon auxiliary electrode, and a printed silver pseudo-reference electrode on a single substrate, providing a compact configuration suitable for portable measurements. The configurations of the working (WE), reference (RE), and auxiliary (AE) electrodes, along with their corresponding electrical contact pads, are illustrated in Figure 1a [20,21].

Electrochemical measurements were performed using a MicruX ECSens BIPOT bipotentiostat (MicruX Technologies, Spain). The device applies controlled potential waveforms and records the resulting current response for voltammetric and amperometric measurements. It operates within a potential range of ±1.5 V, with current measurements spanning from µA to mA and a potential resolution of 250 µV, enabling sensitive detection using screen-printed electrodes. The physical configuration with the inserted electrode is shown in Figure 1b [22].

EC Manager Lite is a graphical user interface (GUI) used to control the MicruX ECSens BIPOT electrochemical sensing system. The software supports experiment setup, execution, visualization, and data management. As shown in Figure 1c, the interface includes modules for (A) protocol and parameter configuration, (B) data visualization and export, (C) real-time current–potential display, and (D) measurement control. Data can be exported in spreadsheet formats (e.g., XLSX) for further analysis, while graphical outputs can be saved as image files (e.g., PNG or JPEG) [22].

Direct copper electrodeposition on commercially available screen-printed carbon electrodes was selected as a simple and reproducible approach compatible with integration into telemedicine and simulation-based workflows, avoiding the complexity of nanostructured sensor fabrication.

### 2.2. Electrode Preparation and Electrochemical Characterization

All reagents were of analytical grade. D-glucose powder was used to prepare standard solutions in phosphate-buffered saline (PBS, pH 10) using an analytical balance and calibrated micropipettes to ensure accurate concentrations. Distilled water was used to rinse the electrodes during the preparation. Solutions were handled under standard laboratory conditions.

Electrode preparation began with the electrochemical activation of the carbon electrodes on the screen-printed three-electrode area (ED-S1PE-C21, comprising working, reference, and auxiliary electrodes). A 70 µL droplet of 1 mM NaOH solution was applied to the working electrode, and activation was performed using an ECSens BIPOT potentiostat. The potential was scanned from 0.0 V to −2.0 V, promoting the formation of oxygen-containing functional groups on the carbon surface, which increases the electroactive area and facilitates subsequent copper nucleation [23,24,25].

Following electrochemical activation, the screen-printed three-electrode area was rinsed with distilled water to remove residual species. Copper electrodeposition was subsequently performed by applying a 70 µL droplet of 0.1 M CuSO_4_ onto the same area. A three-step chronoamperometric deposition protocol was then applied within the same droplet, with potentials sequentially shifted toward more negative values (−0.05 V for 100 s, −0.40 V for 350 s, and −0.60 V for 650 s). This sequence was initially defined based on prior copper-based electrodeposition approaches [26] and subsequently optimized empirically to achieve stable copper deposition on the carbon working electrode through Cu^2+^ ion reduction. The resulting copper-modified surface exhibited behavior consistent with the reported electrocatalytic behavior of copper-based electrodes for non-enzymatic glucose detection [26,27].

The glucose standard solutions were prepared based on the following molar relationship:

m = C × V × M,(1)

where m is the glucose mass, C is the target concentration, V is the solution volume, and M is the molecular weight of D-glucose (180.16 g·mol^−1^). A 100 mM stock solution was prepared by dissolving 36.0 mg of D-glucose in 2 mL of solution. Additional standards (50, 25, 12.5, 6.25, and 3.125 mM) were obtained by serial dilution for electrochemical measurements, enabling the calibration of the sensor response across the studied concentration range [28,29].

Using the same electrochemical setup described above, electrochemical characterization and glucose calibration were performed by cyclic voltammetry to identify the glucose oxidation region, followed by chronoamperometry at a fixed potential. Initially, a 70 µL PBS droplet was placed on the electrode surface. The glucose concentrations were then adjusted by replacing 35 µL of PBS with 35 µL of glucose solution prepared in PBS, maintaining a constant final volume of 70 µL. Accordingly, the final concentration was C_(final)_ = C_(added)_ × (V_(added)_/V_(total)_). The steady-state current was recorded at increasing glucose concentrations, and a calibration curve (current vs. glucose concentration) was obtained to evaluate electrode sensitivity.

The printed silver pseudo-reference electrode integrated into the strip was used consistently for comparative measurements under the same controlled experimental conditions within this proof-of-concept study. The limit of detection (LOD) and limit of quantification (LOQ) were estimated as LOD = 3σ/s and LOQ = 10σ/s, respectively, where σ corresponds to the standard deviation of the steady-state current obtained from repeated measurements under the tested conditions and s is the slope of the calibration curve.

### 2.3. Basic Surface Assessment by SEM-EDX

Scanning electron microscopy coupled with energy-dispersive X-ray spectroscopy (SEM-EDX) was used as a basic morphological and compositional assessment to confirm preferential copper deposition on the modified strip, rather than as an exhaustive physicochemical characterization. Images and spectra were acquired using an Axia ChemiSEM (Thermo Fisher Scientific, Eindhoven, The Netherlands) operated at 20 kV in high-vacuum mode with a Concentric Backscattered (CBS) detector. The analysis focused on three regions of the strip: the working electrode, the auxiliary electrode, and the middle region between them.

### 2.4. Telemedicine Integration in Simulated Gynecology Workflows

The telemedicine platform used was based on a web- and Android-based telemedicine platform (WATP) [30], which evolved from earlier initiatives for rural healthcare and medical training in Ecuador. Previous work validated an initial web-based platform for clinical case discussion and medical education; however, it was limited to browser-based interactions [31]. An AWS-hosted telemedicine deployment was later introduced for rural Cayapas communities and showed limited adoption [32]. To address these limitations, a new WATP was introduced in 2022, incorporating an Android-based telemedicine app (ABTapp, custom-developed version, 2022) for rural practitioners and medical students, with a web platform for specialists and faculty. This platform was developed using an iterative, user-centered design and included offline functionality for teleconsultation in low-connectivity environments [30,33,34].

To address the identified limitations in user engagement, a structured educational implementation strategy was adopted, integrating real and simulated clinical cases within rotating nursing internship programs and final-year undergraduate medical courses. Teleconsultation was implemented through an asynchronous workflow in which clinical cases were generated, submitted, and reviewed by students and supervising faculty as part of a formative assessment process [33,34].

To operationalize this integration, a gynecology simulation scenario was designed following a simulation-based medical education framework, including structured preparation and pre-briefing, and was implemented as a skills-based activity in Simulation Zone 1 at the SIMUEES Clinical Simulation Center [35]. The activity was conducted during the first academic term (April–August 2025) within the gynecology course. Simulated blood samples with five predefined glucose concentrations were prepared to reproduce clinical variability. The students handled the samples under standard precautions using gloves and a micropipette (70 µL), applied each sample to a previously modified working electrode, and acquired the electrochemical signal using the portable potentiostat described in the experimental section.

The measured current was converted into glucose concentration (mg/dL) using a spreadsheet-based calibration template derived from the experimental linear calibration model. The template was implemented as a standalone tool in which the steady-state current obtained from chronoamperometric measurements was manually entered, and the corresponding glucose value was automatically calculated. This value was then entered into the teleconsultation form within the telemedicine platform as part of a simulated gynecology clinical case. The activity was conducted as a supervised formative assessment, requiring students to interpret the obtained value in the clinical context and justify their diagnostic decisions during the teleconsultation process.

After the simulation activity, implementation was evaluated using a structured survey administered via the QuestionPro platform (QuestionPro Inc., cloud-based survey software, version used during the study period, accessed in 2025) to assess perceived usability and formative value of the simulation scenario. Students and supervising faculty completed the questionnaire, providing feedback on the integration of electrochemical measurements within the teleconsultation workflow and its relevance for clinical learning.

This study was conducted in a simulation-based educational setting and did not involve real patients or human biological samples. Participants included 24 final-year undergraduate medical students enrolled in a gynecology course and 2 supervising gynecology faculty members. All participants provided written informed consent, and participation was voluntary. The study was conducted under the institutional research project “Efectividad de la telemedicina como herramienta de simulación en el desarrollo de competencias de diagnóstico a distancia en estudiantes de medicina,” approved by the Research Center (CIN) of Universidad Espíritu Santo (UEES), Ecuador [36]. According to institutional policies, this type of educational study without real patient involvement does not require formal biomedical ethics committee approval.

## 3. Results

### 3.1. Surface and Electrochemical Characterization of the Cu-Modified Electrode

**Figure 2:**
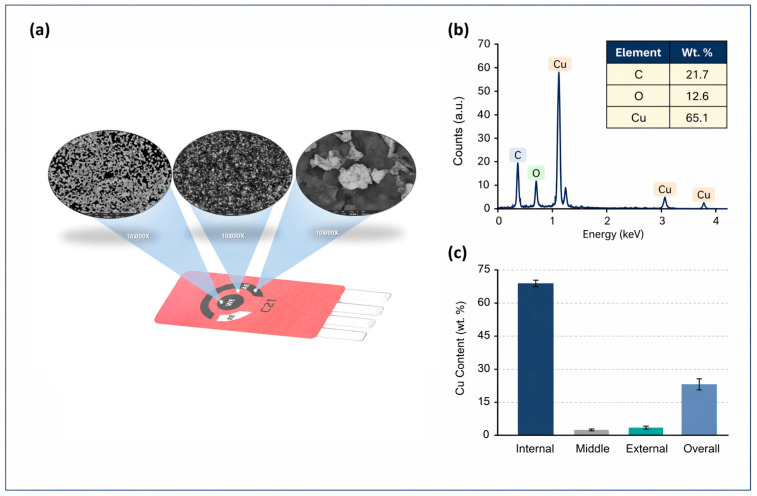
SEM–EDX characterization of the Cu-modified electrode: (**a**) SEM images at 10,000×, (**b**) EDX spectrum of the working electrode (WE), and (**c**) quantitative Cu content.

SEM images and EDX spectra were acquired using an Axia ChemiSEM operated at 20 kV in high-vacuum mode with a Concentric Backscattered (CBS) detector. Figure 2a presents SEM micrographs obtained at 10,000× magnification, showing the internal region corresponding to the working electrode (WE), the middle region between the WE and the auxiliary electrode (AE), and the external region corresponding to the AE area. The internal region exhibited a heterogeneous particulate/nodular morphology, clearly differentiated from the more uniform appearance observed in the middle region and from the localized irregular aggregates observed in the external region. These observations provide basic morphological evidence of surface differences across the strip after copper electrodeposition.

The compositional analysis was focused on the working electrode (WE) region, as it represents the active sensing area of the device. The representative EDX spectrum shown in Figure 2b indicates copper enrichment on the analyzed WE surface, with a dominant Cu signal together with carbon (C) and oxygen (O), attributed to the carbon-based substrate and surface oxidation. Quantitative analysis of this region showed that copper was the predominant element (65.1 wt.%), followed by carbon (21.7 wt.%) and oxygen (12.6 wt.%). Figure 2c presents the corresponding quantitative Cu content in the analyzed WE region in a bar chart format, highlighting its predominance relative to the other detected elements. These results support preferential copper deposition in the WE area, consistent with the morphological differences observed in Figure 2a.

**Figure 3:**
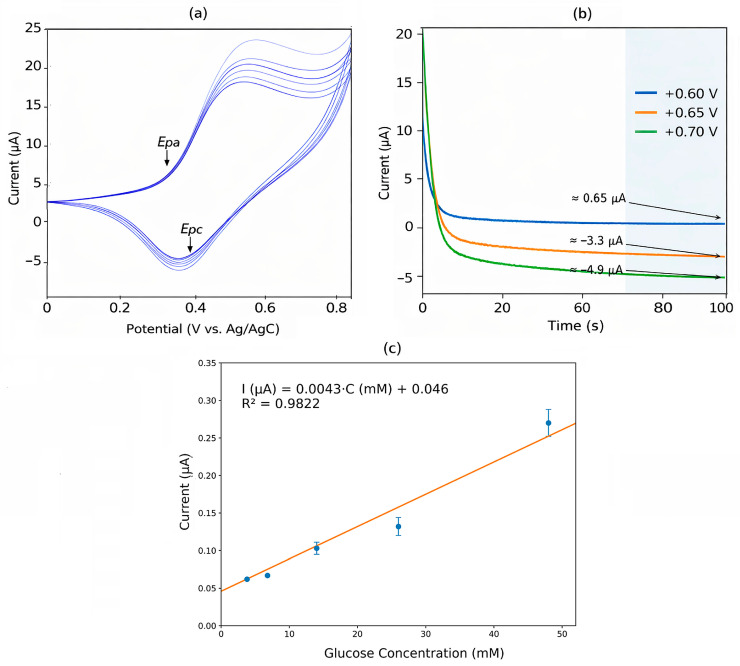
Electrochemical characterization of the Cu-modified electrode for glucose detection: (**a**) cyclic voltammetry, (**b**) chronoamperometric response at different applied potentials, and (**c**) linear calibration curve.

Figure 3 shows the electrochemical response of the Cu-modified working electrode (WE) toward glucose detection. The cyclic voltammograms in Figure 3a exhibit defined anodic (Epa) and cathodic (Epc) peaks. The chronoamperometric responses in Figure 3b, recorded at +0.60, +0.65, and +0.70 V, display distinct current levels that stabilize over time. The calibration curve in Figure 3c demonstrates a linear relationship between current and glucose concentration within the evaluated range, described by I (µA) = 0.0043·C (mM) + 0.046 with R^2^ = 0.9822.

A linear relationship between glucose concentration and steady-state current was observed in the range of 3.125–50 mM (R^2^ = 0.9822), while a deviation from linearity was detected at 100 mM. The calibration slope was 0.00433 μA·mM^−1^, corresponding to an area-normalized sensitivity of 0.061 μA·mM^−1^·cm^−2^ for an electrode area of 0.071 cm^2^. The limits of detection (LOD) and quantification (LOQ) were estimated as 1.39 mM and 4.62 mM, respectively.

### 3.2. Results of Telemedicine Integration in Simulated Gynecology Workflows

**Figure 4:**
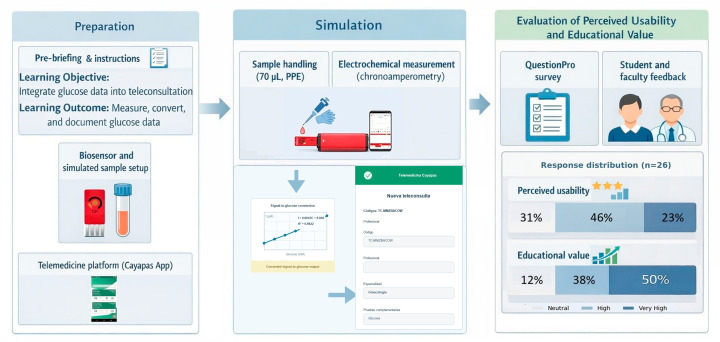
Implemented workflow for electrochemical glucose data integration into a simulated gynecological teleconsultation, including sample handling, electrochemical measurement, external signal-to-glucose conversion, and manual entry into the telemedicine platform.

Figure 4 summarizes the simulation-based workflow for integrating electrochemical glucose measurement into teleconsultation and evaluating its perceived usability and educational value. Prepared glucose sample solutions were used, and each participant completed the measurement process, documented the results, and submitted a teleconsultation through the Cayapas platform (ABTapp, custom-developed version, 2022). Among the 26 participants (*n* = 26), the evaluation showed high acceptance of the workflow, with 69% reporting high or very high perceived usability and 88% reporting high or very high educational value. These results support the feasibility of this integration as a simulation-based telemedicine approach with strong perceived learning benefits.

## 4. Discussion

The copper-modified carbon electrode exhibited a concentration-dependent amperometric response in PBS (pH 10), with linearity observed between 3.125 and 50 mM (R^2^ = 0.9822). The use of PBS at pH 10 was intentionally selected to enable stable electrochemical activity of the copper-modified electrode under controlled conditions and should not be interpreted as representative of physiological environments. A slight deviation from linearity was observed at 100 mM glucose, which may be attributed to diffusion limitations or partial surface saturation at higher glucose concentrations. Cyclic voltammetry and chronoamperometry confirmed the electrochemical activity of the copper-modified surface at the selected operating potential of +0.60 V. The low relative standard deviation (<4% RSD) further indicated good repeatability under the tested conditions. These findings support the use of direct copper modification of carbon-based electrodes for non-enzymatic glucose sensing under buffered conditions [26,27]. These findings should be interpreted within the controlled alkaline conditions used in this proof-of-concept study and not as evidence of direct applicability to physiological samples.

Rather than maximizing analytical performance through complex nanostructured architectures [37,38,39], the present study adopted direct copper electrodeposition on commercially available screen-printed carbon electrodes [26,27] as a practical strategy compatible with the intended simulation-based workflow. SEM-EDX provided basic morphological and compositional support for this approach by confirming preferential yet heterogeneous copper deposition on the working electrode. In this context, the main contribution of the study is not the development of a clinically optimized glucose sensor, but the functional integration of a simple electrochemical sensing module into a teleconsultation-oriented simulation workflow. In this context, the value of the proposed sensor lies not only in glucose detection but also in its straightforward preparation, reproducible implementation, and compatibility with educational and teleconsultation-oriented settings, where operational simplicity is essential for system integration [40,41,42].

Furthermore, the usability and educational evaluation results (*n* = 26) showed favorable acceptance of the proposed workflow, with 69.23% of the participants reporting positive usability ratings and 88.47% indicating strong educational value. Neutral usability responses (26.92%) highlighted opportunities to refine the usage protocol and further improve the user experience and learning outcomes in this and other medical training courses. One potential improvement involves standardizing the interaction steps and providing brief user guidance prior to the simulation to enhance consistency and ease of use [41].

Within the implemented workflow, signal-to-glucose conversion was performed using an external spreadsheet-based calibration template, and the resulting glucose value was then manually entered into the teleconsultation record. Although this configuration was sufficient for the educational objective of interpreting electrochemical data within a simulated scenario, future versions should embed automated signal conversion directly into the software interface to enable real-time display and reduce operator-dependent steps.

Analytical limitations should be acknowledged. The present study did not include interference analysis, validation in real biological samples, or operation under physiological pH conditions, and the pseudo-reference electrode was used only for comparative measurements within a controlled setup. These aspects reflect the scope of this work as an engineering and educational proof-of-concept rather than a clinically validated sensing platform. Future work will address these points through validation in real samples, interference studies, and evaluation under physiologically relevant conditions.

From an educational perspective, the implementation was conducted within a single academic period with a limited cohort (*n* = 26), enabling controlled deployment and feasibility assessment. Future studies will extend validation to larger cohorts and multiple academic periods, incorporating more realistic simulation scenarios and structured evaluation strategies to strengthen the educational validity of the proposed workflow. Overall, the proposed approach demonstrates the feasibility of integrating electrochemical glucose sensing into a teleconsultation-based training environment under controlled simulated conditions, providing a practical foundation for further educational validation and staged technical refinement.

## 5. Conclusions

This study demonstrated the integration of a portable copper-modified non-enzymatic glucose sensor into a telemedicine platform within a simulation-based medical education framework. The proposed workflow encompasses sensor preparation, electrochemical measurement, and the transformation of signals into clinically interpretable glucose values for teleconsultation. SEM-EDX characterization provided basic confirmation of preferential copper deposition on the working electrode, supporting the selected surface modification strategy within the scope of this proof-of-concept. Therefore, the use of a portable electrochemical glucose sensor, simulated samples, and a gynecological teleconsultation simulation workflow provided a controlled and safe environment, enabling reproducible implementation within structured training scenarios.

In this context, the positive usability and educational perceptions observed among participants support the practical feasibility of this approach in simulation-based learning. The proposed workflow can be adapted to similar gynecological scenarios and extended to other medical training contexts where glucose monitoring is clinically relevant, supporting its replication across simulation centers.

In summary, the study demonstrates the feasibility of integrating an electrochemical sensing step into a teleconsultation workflow for educational use under controlled simulated conditions. Before considering translation toward real clinical applications, further analytical optimization, interference testing, operation under physiologically relevant conditions, and validation in real samples will be required.

## Data Availability

Raw electrochemical data supporting the findings of this study, including cyclic voltammetry and chronoamperometric measurements, are available from the corresponding author upon reasonable request. SEM-EDX images and compositional outputs are also available from the corresponding author upon reasonable request. No publicly archived datasets were generated in this study.

## Abbreviations

The following abbreviations are used in this manuscript:

Ag	Silver
Ag/AgCl	Silver/Silver Chloride
AE	Auxiliary Electrode
CNTs	Carbon Nanotubes
CuO	Copper(II) Oxide
CV	Cyclic Voltammetry
LOD	Limit of Detection
LOQ	Limit of Quantification
MOF	Metal–Organic Framework
NaOH	Sodium Hydroxide
PBS	Phosphate-Buffered Saline
RE	Reference Electrode
RSD	Relative Standard Deviation
WE	Working Electrode
CA	Chronoamperometry
SPCE	Screen-Printed Carbon Electrode
GUI	Graphical User Interface
R^2^	Coefficient of Determination
mg/dL	Milligrams per Deciliter
